# MCPIP1-mediated NFIC alternative splicing inhibits proliferation of triple-negative breast cancer via cyclin D1-Rb-E2F1 axis

**DOI:** 10.1038/s41419-021-03661-4

**Published:** 2021-04-06

**Authors:** Fengxia Chen, Qingqing Wang, Xiaoyan Yu, Ningning Yang, Yuan Wang, Yangyang Zeng, Zhewen Zheng, Fuxiang Zhou, Yunfeng Zhou

**Affiliations:** 1 Hubei Key Laboratory of Tumor Biological Behaviors, Zhongnan Hospital of Wuhan University, Wuhan, China; 2 Department of Radiation and Medical Oncology, Zhongnan Hospital of Wuhan University, Wuhan, China; 3 Hubei Cancer Clinical Study Center, Zhongnan Hospital of Wuhan University, Wuhan, China

**Keywords:** Cancer, Cancer genetics

## Abstract

Triple-negative breast cancer (TNBC) is the most aggressive subtype with the worst prognosis and the highest metastatic and recurrence potential, which represents 15–20% of all breast cancers in Chinese females, and the 5-year overall survival rate is about 80% in Chinese women. Recently, emerging evidence suggested that aberrant alternative splicing (AS) plays a crucial role in tumorigenesis and progression. AS is generally controlled by AS-associated RNA binding proteins (RBPs). Monocyte chemotactic protein induced protein 1 (MCPIP1), a zinc finger RBP, functions as a tumor suppressor in many cancers. Here, we showed that MCPIP1 was downregulated in 80 TNBC tissues and five TNBC cell lines compared to adjacent paracancerous tissues and one human immortalized breast epithelial cell line, while its high expression levels were associated with increased overall survival in TNBC patients. We demonstrated that MCPIP1 overexpression dramatically suppressed cell cycle progression and proliferation of TNBC cells in vitro and repressed tumor growth in vivo. Mechanistically, MCPIP1 was first demonstrated to act as a splicing factor to regulate AS in TNBC cells. Furthermore, we demonstrated that MCPIP1 modulated *NFIC* AS to promote CTF5 synthesis, which acted as a negative regulator in TNBC cells. Subsequently, we showed that CTF5 participated in MCPIP1-mediated antiproliferative effect by transcriptionally repressing cyclin D1 expression, as well as downregulating its downstream signaling targets p-Rb and E2F1. Conclusively, our findings provided novel insights into the anti-oncogenic mechanism of MCPIP1, suggesting that MCPIP1 could serve as an alternative treatment target in TNBC.

## Introduction

Breast cancer is the most common malignancy and the fourth leading cause of cancer-related deaths among women in China, accounting for 19.9% of all cancer diagnosed and 9.9% of all cancer-associated deaths in females in 2020^1^. Triple-negative breast cancer (TNBC), defined by a lack of expression of estrogen receptor (ER), progesterone receptor (PR) expression, and human epidermal growth factor receptor 2 (HER2), is the most aggressive subtype among breast cancers^2,3^. This subtype represents 15–20% of all breast cancers in Chinese women^4^ and is more common in patients with younger age, African-American race, and BRCA1 germline mutations^5^. TNBC patients often have poor clinical outcomes due to the high risk of early relapse and visceral metastases within 5 years^6^. The 5-year overall survival (OS) rate is about 80% in Chinese women^7,8^. Owing to the absence of effective targets, systematic chemotherapy remains the mainstay of TNBC patients^9^. However, the efficacy of second-line chemotherapy is limited by low response rates and short progression-free survival (PFS), leading to unchanged OS among TNBC patients over the past two decades^10,11^. Therefore, effective therapeutic targets are urgently identified.

Alternative splicing (AS) is a major mechanism for generating multiple structurally and functionally different proteins from a single gene, greatly expanding proteome diversity^12^. Accumulating evidence has demonstrated that dysregulated AS contributed to malignant diseases, including cancer, especially breast cancer^13–15^. Cancer cells often display aberrant AS profiles, which are associated with apoptosis^16^, angiogenesis^17^, migration^18^, and drug resistance^19^. Nowadays, growing evidence indicates that aberrations in AS are considered a molecular hallmark of cancer^20^. These findings highlighted the possibility that manipulation of AS might provide a potential target for cancer treatment.

The process of AS is generally controlled by many RNA-binding proteins (RBPs) which interact with specific RNA sequences to either promote or repress splicing^21,22^. It has been reported that these AS-associated RBPs were often dysregulated in cancer and differently expressed between tumor and normal tissues^21^. Monocyte chemotactic protein induced protein 1 (MCPIP1), alias Regnase-1 encoded by the *zc3h12a* gene, is a conserved zinc finger RBP^23^. MCPIP1 has pleiotropic activity due to its multi-domain structure, a zinc finger motif mediated DNA and RNA binding, PIN domain exhibiting RNase activity, and ubiquitin‐binding domain possessing deubiquitinase activity^24^. MCPIP1 acts mainly as a ribonuclease that negatively regulates inflammation^24^, controls immune response^25^, suppresses microRNA biosynthesis^26^, and degrades some viral RNAs^27^. Recent studies have shown that MCPIP1 was downregulated and functioned as a tumor suppressor in several cancers, including breast cancer^28^, clear‐cell renal cell carcinoma (ccRCC)^29^, neuroblastoma^30^, and osteosarcoma^31^. Furthermore, MCPIP1 exhibited antineoplastic roles in an RNase-dependent manner in these tumors. However, whether MCPIP1 exerts an effective antitumor effect by regulating AS in TNBC is unclear.

This study aimed to identify MCPIP1-regulated alternative splicing events (ASEs) and investigate the contributions of these ASEs to the cell cycle progression and proliferation in TNBC cells both in vitro and in vivo. Here, we found that MCPIP1 was downregulated in TNBC tissues and cell lines compared to normal tissues and cell lines. Moreover, high MCPIP1 level was associated with prolonged OS in TNBC patients. Importantly, we first showed that MCPIP1 regulated AS in TNBC cells. Additionally, we demonstrated that MCPIP1 modulated NFIC AS to promote the production of CTF5, which was responsible for MCPIP1-mediated antiproliferative effect by suppressing the cyclin D1 transcription in TNBC.

## Materials and methods

### Tissue microarray

TNBC tissue microarray containing 80 pairs of TNBC and corresponding normal tissues (cat. no. BRC1601) was obtained from Wuhan Baiqiandu Technology (Wuhan, China). Tumor staging were determined according to the Eighth Edition AJCC Cancer Staging for breast cancer. The details of clinicopathological characteristics are listed in Supplementary Table 1.

### Immunohistochemistry

Immunohistochemistry (IHC) assay was carried out according to previous description^32^. Five random high-power fields were detected for each slide. Quantifications of the proteins were calculated by Image J software.

### Cell lines and culture

The TNBC cell lines (Hs578T, BT549, MDA-MB-231, MDA-MB-157 and MDA-MB-468), human immortalized mammary epithelial cell line (MCF10A), and 293 T cells were purchased from Procell Life Science & Technology (Wuhan, China). MDA-MB-231, MDA-MB-157, MDA-MB-468, Hs578T, BT549, and 293 T cells were cultured in DMEM (Gibco, USA) medium containing 10% FBS (Wisent, Canada). MCF10A cells were grown in complete DMEM/F12 medium (Procell Life Science & Technology). All cells were maintained at 37 °C with 5% CO_2_.

### Plasmids construction and cell transfection

The pcDNA3.1-MCPIP1, pcDNA3.1-CTF5, pEZ-M02-cyclin-D1 overexpression plasmids and luciferase reporter plasmids incorporating cyclin D1 promoter fragment were constructed by GeneCopoeia (Guangzhou, China). Minigenes were constructed by ABlife (Wuhan, China). siRNA targeting MCPIP1 was purchased from GenePharma (Shanghai, China). Three different MCPIP1 siRNA duplexes were tested, and siRNA#1 was used in the following experiments (Supplementary Fig. S1A). Sequences of siMCPIP1 were shown in Supplementary Table 2. Plasmids and siRNA were transfected into cells with Lipofectamine 3000 (Invitrogen, USA) according to the manufacturer’s protocol. MDA-MB-231 cells transfected with the MCPIP1 overexpression plasmid were then selected with G418 (Gibco, USA) for approximately 4 weeks. Surviving cells were used as stable mass transfectants.

### RNA extraction, reverse transcription, quantitative real-time PCR (qRT-PCR) and agarose gel electrophoresis

Trizol reagent (Invitrogen, USA) was used for total RNA extraction and complementary DNA (cDNA) was then synthesized using the TRUEscript RT Kit (Aidlab Biotechnology, China) according to manufacture’s protocol. qRT-PCR were performed with 2 × Sybr Green qPCR Mix (Aidlab Biotechnology, China) according to manufacture’s protocol. The relative mRNA expression level was quantitated with the 2^−^^∆∆CT^ method. Agarose gel electrophoresis was performed by ABlife (Wuhan, China). *GAPDH* served as an internal control. Primer sequences are shown in Supplementary Table 2.

### Western blotting

Western blot assay were conducted according to previous description^32^. The primary antibodies used include the following: anti-MCPIPI (#GTX110807, GeneTex, 1:1500), anti-cyclin D1 (#A19038, ABclonal, 1:1000); anti-RB (#25628–1-AP, Proteintech, 1:1000), anti-pRB(Ser780) (#9307, Cell Signaling Technology, 1:1000), anti-E2F1 (#12171–1-AP, Proteintech, 1:1000), anti-Flag-Tag (#T0003, Affinity Biosciences, 1:5000), anti-GAPDH (#60004–1-Ig, Proteintech, 1:5000), anti-β-actin (#60008-1-Ig Proteintech, 1:1000). The secondary antibodies used include HRP-conjugated goat anti-mouse antibody (#SA00001-1, Proteintech, 1:10,000) and HRP-conjugated goat anti-rabbit antibody (#SA00001-2, Proteintech, 1:10,000). The bands were detected with ECL reagent (Advansta, USA). Image J software was used to calculate the protein expression levels.

### Cell Counting Kit-8 (CCK-8) assay

MDA-MB-231 and MDA-MB-157 cells were transiently transfected with the corresponding plasmids and siRNAs. The next day the transfected cells (3 × 10^3^/well) were plated in 96-well plates in triplicates. Cell viability was assessed at 24, 48, 72 and 96 h, respectively. CCK-8 reagent (10 μL/well) was added to each well and incubated at 37 °C in dark for 2 h. The absorbance at 450 nm was examined with the SpectraMax Absorbance Reader (Molecular Devices, USA).

### Colony formation assay

The transfected MDA-MB-231 and MDA-MB-157 cells (200/well) were seeded in 6-well plates and then maintained for 14 days. Then, colonies were first fixed using 4% paraformaldehyde (ServicBiotech) and then stained using 0.1% crystal violet. Cell colonies with more than 50 cells were calculated.

### 5-Ethynyl-20-deoxyuridine (EdU) assay

Approximately 1 × 10^4^ transfected MDA-MB-231 and MDA-MB-157 cells per well were seeded in 96-well plates and then incubated for 24 h. The EdU assay was conducted with Cell-Light^TM^ EdU Kit (RiboBio, China) according to the manufacturer’s protocol. Five fields of each well were randomly chosen for observation under fluorescence microscopy (Nikon, Japan).

### Cell cycle assay

The transfected MDA-MB-231 and MDA-MB-157 cells were harvested, and then mixed with propidium iodide (Multi Sciences, China) according to the manufacturer’s instructions. Then, cell cycle were detected immediately using flow cytometry (Cytoflex, Beckman, USA) with CytExpert software (Beckman, USA).

### RNA sequencing and data analysis

RNA sequencing (RNA-seq) and data analysis were conducted by ABlife (Wuhan, China). The prepared library was subjected to RNA-seq using an Illumina HiSeq X Ten system (Illumina, China) to collect 150 nt paired-end sequencing. Next, differentially expressed genes (DEGs) were screened out using the edgeR package by meeting the threshold of fold change ≥2 or ≤0.5, false discovery rate (FDR) <0.05. Subsequently, the ASEs and regulated ASEs (RASEs) were analyzed as previously described^33^. Briefly, ten types of ASEs were detected in each sample based on splice junction reads, including cassette exon, exon skipping (ES), intron retention (IR), alternative 3’ splice site (A3SS), alternative 5′ splice site (A5SS), mutually exclusive exons(MXE), mutually exclusive 5’ UTRs (5pMXE), mutually exclusive 3′ UTRs (3pMXE), A5SS&ES, and A3SS&ES. Student’ s *t*-test was performed to evaluate MCPIP1-regulated ASEs via calculating the ratio alteration of ASEs. *P* < 0.05 was considered statistically significant for detection of RASEs between the two compared samples. The ratio of ASEs in RNA-seq was calculated using the formula: alternative junction reads/(alternative junction reads + model junction reads), while the ratio of ASEs in qRT-PCR was calculated using the formula: alternative splicing transcript level/model transcript level.

### Improved RNA immunoprecipitation sequencing and data analysis

Improved RNA immunoprecipitation (iRIP) and data analysis were performed by ABlife (Wuhan, China), and the detailed procedures were as previously described^34^. cDNA libraries were established, and then Illumina HiSeq X Ten system was used for high-throughput sequencing of the cDNA libraries for 150 nt paired-end sequencing. Only peaks higher than those of the random max peaks (*p*-value < 0.05) were included. The immunoprecipitation peaks that overlapped with the input peaks were excluded. The target genes of immunoprecipitation were identified by the peaks, and finally the binding motifs of immunoprecipitation protein were called using the HOMER software.

### Functional enrichment analysis

To comprehensively assess the biological functions of regulated alternative genes (RASGs), Gene Ontology (GO) terms and Kyoto encyclopedia of genes and genomes (KEGG) pathways analysis were performed with the KOBAS 2.0 server.

### Immunofluorescence assay

Flag-tagged CTF5 plasmid was transiently transfected into MDA-MB-231 and MDA-MB-157 cells and incubated for 24 h. The transfected cells (1 × 10^4^/well) were then plated on the coverslips in a 12-well plate and incubated for another 24 h. Immunofluorescence assay was conducted as previously described^35^. The primary antibody anti-Flag-Tag (#T0003, Affinity, 1:200) and a Cy3-conjugated secondary goat anti-rabbit antibody (#BA1032, Boster, 1:100) were used in this study. Images were obtained on a fluorescence microscope (Nikon, Japan).

### Chromatin immunoprecipitation assay

Flag-tagged CTF5 plasmid or empty vector was transiently transfected into MDA-MB-231 and MDA-MB-157 cells. Cells were harvested for Chromatin immunoprecipitation (ChIP) using the Pierce Agarose ChIP Kit (Thermo, USA) according to the manufacturer’s protocol. The primary antibody used in this experiment was flag antibody (#F1804, Sigma-Aldrich, 1:1000). Primer sequences used for ChIP are shown in the Supplementary Table 2.

### Luciferase reporter assay

293 T cells were co-transfected with 100 ng firefly luciferase and CTF5 plasmid/vector and incubated for 48 h. Then, the luciferase activity was detected with firefly luciferase reporter gene assay kit (Beyotime Biotechnology, China) using a luminometer (Omega, USA).

### Tumor xenografts experiments

Animal experiments were approved by the Institutional Animal Care and Use Committee of Wuhan University. Ten four-week-old female BALB/c nude mice were randomly divided into two groups (*n* = 5/group) and then subcutaneously inoculated with MDA-MB-231 cells (2 × 10^7^) stably transfected with MCPIP1-overxpression plasmids or control vector, respectively. Then, the mice were maintained for 21 days and tumor volume was monitored every 3 days. Three weeks later, the mice were euthanized to harvest the xenografts for immunohistochemistry and qRT-PCR.

### Statistical analysis

Data was analyzed using SPSS 20.0 (Chicago, USA) software and GraphPad Prism 8.0 (San Diego, USA), and was presented as the mean ± standard deviation (SD). Data was analyzed using student’s *t*-test for two groups or one-way ANOVA for multiple groups. The log-rank test was performed to evaluate the statistical significance of OS. The Chi-square test was performed to analyze the association between clinicopathological parameters of TNBC patients and MCPIP1 expression. *P* < 0.05 was considered statistically significant.

## Results

### MCPIP1 is down-regulated in TNBC tissues and cell lines, and MCPIP1 expression level is positively associated with the prognosis in TNBC patients

**Fig. 1:**
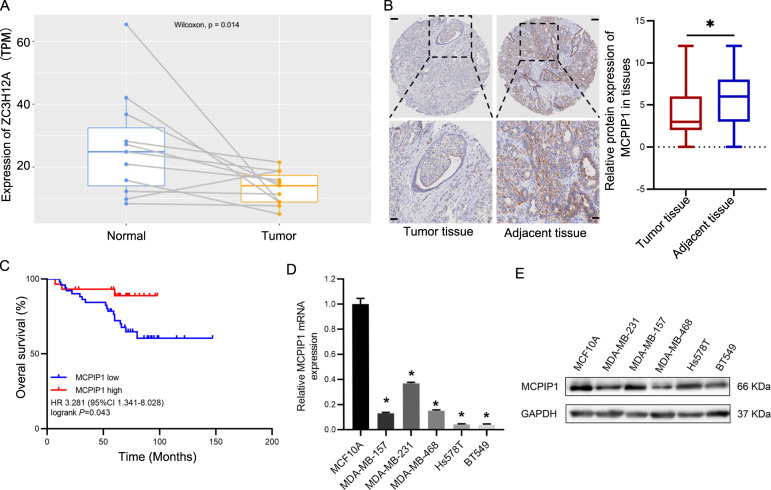
The expression of MCPIP1 in TNBC tissues and cell lines and association with survival. **A** Analysis of MCPIP1 mRNA levels from TCGA database, including 11 TNBC tumor and paired normal tissues. **B** The expression level of MCPIP1 is detected by IHC in 80 pairs of TNBC tissues and corresponding adjacent normal mammary tissues. Scale bars, 50 and 200 μm. **C** Association between MCPIP1 expression levels and overall survival in 80 patients with TNBC using Kaplan–Meier survival curve. **D**, **E** The relative expression of MCPIP1 is detected by qRT-PCR and western blot in MCF10A, MDA-MB-157, MDA-MB-231, MDA-MB-468, Hs578T and BT549 cells. *GAPDH* is used as endogenous control. Error bars represent the mean ± SD from three independent experiments. *P < 0.05.

It was recently reported that MCPIP1 expression was decreased in breast cancers and that low MCPIP1 level was correlated with poor survival in breast cancer patients^28^. However, previous studies primarily focused on breast cancer without taking its subtype into consideration, because breast cancer is a highly heterogeneous disease that possesses a complex pathogenesis, various biological behaviors and therapeutic responses. Accordingly, we first analyzed MCPIP1 expression levels using the data available from the Cancer Genome Atlas (TCGA) database, which revealed that MCPIP1 expression was markedly downregulated in TNBC tissues compared to the corresponding normal samples (Fig. 1A). Subsequently, we examined MCPIP1 expression levels in 80 pairs of TNBC tissues and paired normal breast tissues and observed similar results (Fig. 1B). Moreover, it was shown that high MCPIP1 levels were correlated with prolonged OS (hazard ratio, 3.281; 95% confidence interval, 1.341–8.028; *P* = 0.043) (Fig. 1C). However, MCPIP1 expression levels were not significantly associated with the clinicopathological characteristics, including age, grade, tumor size, lymph node status, and TNM staging (Supplementary Table 1). In addition, we found that MCPIP1 expression was also reduced in TNBC cell lines (MDA-MB-157, MDA-MB-231, MDA-MB-468, Hs578T, and BT549) compare with human immortalized breast epithelial cells (MCF10A) (Fig. 1D, E). Taken together, these data demonstrated that MCPIP1 was downregulated in TNBC tissues and cell lines, and high MCPIP1 expression was highly predictive of better prognosis in TNBC patients.

### Overexpression of MCPIP1 suppresses cell proliferation and induces cell cycle arrest in TNBC cells both in vitro and in vivo

**Fig. 2:**
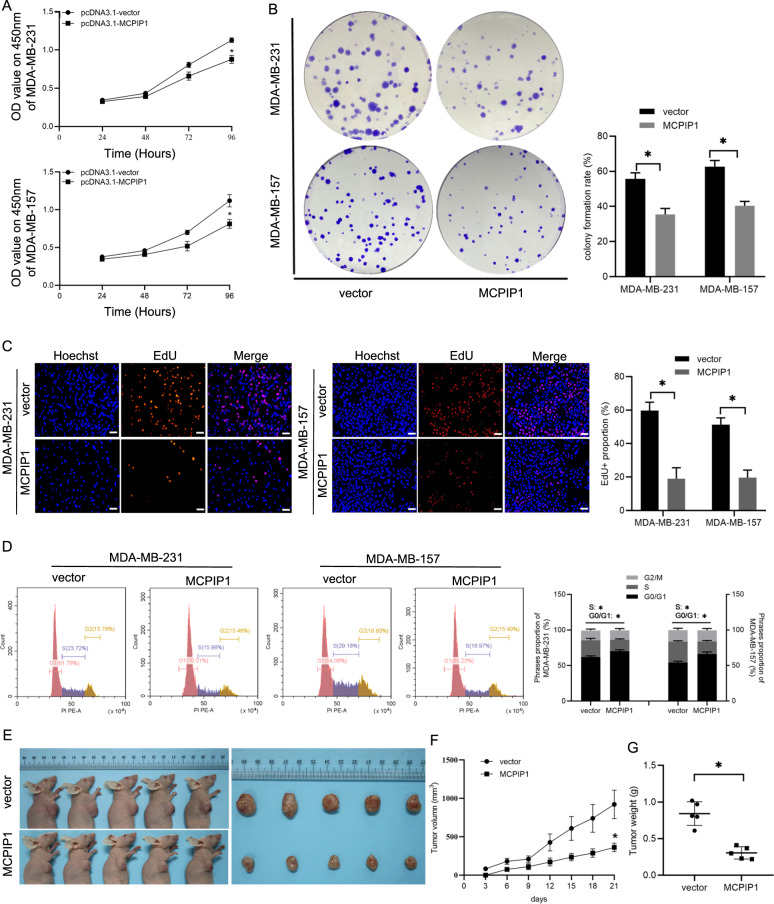
MCPIP1 inhibits proliferation and cell cycle progression of TNBC cells. **A** MDA-MB-231 and MDA-MB-157 cells are transfected with the control vector or MCPIP1 overexpression plasmids. CCK8 assay is performed to examine cell viability at 24, 48, 72, and 96 h. **B** Colony formation is performed after the indicated transfections. **C** EdU assay is performed following the indicated transfections to detect cell proliferation. Scale bars, 50 μm. **D** Cell cycle is detected using flow cytometry after the indicated transfections. **E** Representative pictures of the mouse subcutaneous xenograft models and xenografts extracted from the two treatment groups after euthanasia. **F**, **G** The tumor growth and weight are decreased in MCPIP1-overexpresses group in comparison with control vector group. Error bars represent the mean ± SD from three independent experiments. *P < 0.05.

To investigate the effect of MCPIP1 on TNBC cell proliferation, MCPIP1 overexpression plasmid was transfected into MDA-MB-231 and MDA-MB-157 cells (Supplementary Fig. 1B). CCK-8 assay showed that enforced expression of MCPIP1 significantly suppressed the viability of TNBC cells (Fig. 2A). Colony formation assay and EdU assay confirmed that MCPIP1 overexpression suppressed TNBC cell proliferation (Fig. 2B, C). It is well known that cell cycle arrest often results in decreased cell proliferation; therefore, we examined the alterations in cell cycle after overexpression of MCPIP1 in TNBC cells. Cell cycle analysis showed that MCPIP1 over-expressing TNBC cells exhibited a higher proportion of cells within the G0/G1 stage and lower in the S stage (Fig. 2D). To better clarify MCPIP1 anti-proliferation activity, siRNA against MCPIP1 was transfected into TNBC cells to evaluate the impact on cell proliferation and cell cycle (Supplementary Fig. 1B). In contrast, silencing MCPIP1 significantly increased TNBC cell proliferation and promoted cell cycle transition from the G0/G1 stage to the S stage (Supplementary Fig. 2).

We further explored the influence of MCPIP1 overexpression on tumor growth in vivo. MDA-MB-231 cells stably transfected with MCPIP1 overexpression plasmid or the vector were subcutaneously inoculated into female BALB/c nude mice. As expected, enforced expression of MCPIP1 hindered tumor growth and reduced tumor volume and tumor weight (Fig. 2E–G). Collectively, these results suggested that MCPIP1 inhibited cell proliferation and prevents cell cycle progression of TNBC cells.

### Identification of MCPIP1-dependent AS events in TNBC cells

**Fig. 3:**
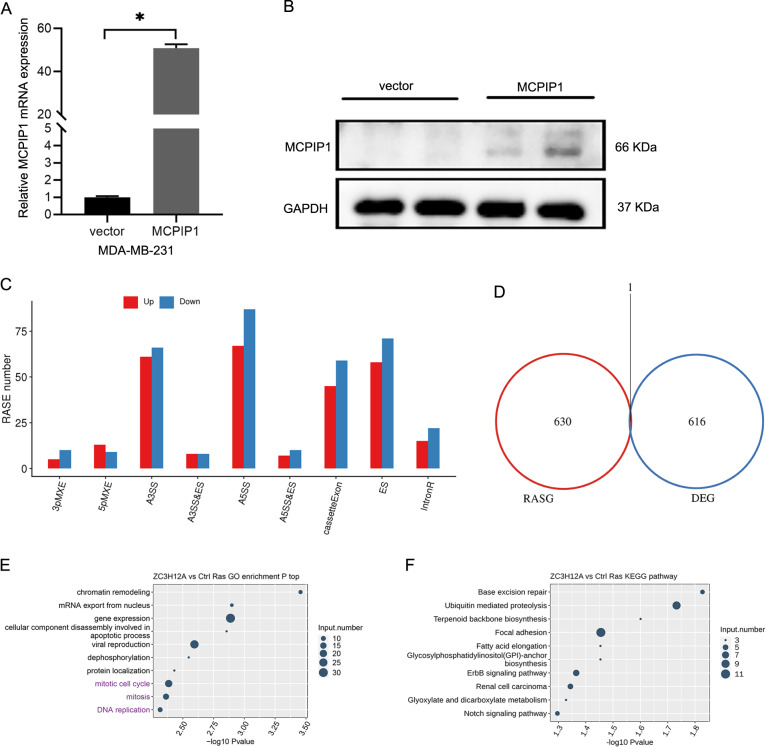
Identification and functional enrichment analysis of MCPIP1-regulated ASEs. **A**, **B** Quantification of MCPIP1 overexpression using qRT-PCR and western blot. *GAPDH* is used as endogenous control. Error bars represent the mean ± SD from three independent experiments. *P < 0.05. **C** Nine AS types regulated by MCPIP1. **D** The result of overlap analysis between MCPIP1-regulated DEGs and RASGs. **E**, **F** Top 11 GO biological processes and KEGG pathways enriched by RASGs are shown in bubble plots.

Considering that MCPIP1 is a multifunctional RBP, we hypothesized that MCPIP1 acted as a splicing factor in TNBC. To identify MCPIP1-regulated ASEs in TNBC cells, we explored the influences of MCPIP1 overexpression on the AS profiles in MDA-MB-231 cells using RNA-seq analysis (Fig. 3A, B). A total of 762 ASEs were associated with MCPIP1 expression (Supplementary Table 3) in MDA-MB-231 cells, especially A3SS, A5SS, cassette exon, and ES (Fig. 3C). To exclude the possibility that the alterations in ASEs could be simply attributed to transcriptional regulation, we performed an overlap analysis between MCPIP1-regulated DEGs and alternative splicing genes (RASGs). The results indicated that there were no significant changes in transcriptional levels in RASGs (Fig. 3D). Further functional analysis revealed that these RASGs were mainly enriched in the mitotic cell cycle, DNA replication, ErbB signaling pathway, and Notch signaling pathway (Fig. 3E, F), indicating that the impact of MCPIP1 on AS was significantly correlated with the regulation of cell cycle progression and proliferation in TNBC^36,37^. In summary, these data demonstrated that MCPIP1 extensively regulated ASEs in TNBC cells.

### MCPIP1 promotes the production of CTF5 by regulating the AS of NFIC

**Fig. 4:**
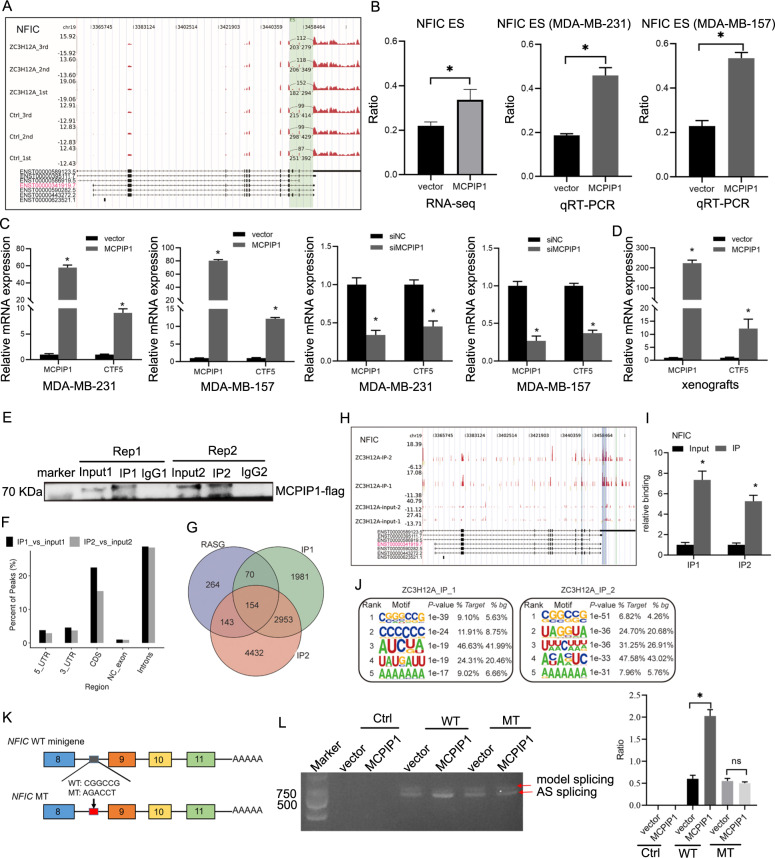
MCPIP1 binds to *NFIC* pre-mRNA and increases exon 9 and 10 skipping to promote the production of CTF5. **A** IGV-sashimi plot shows an exon skipping of NFIC. **B** RNA-seq quantification and qRT-PCR validation of ASEs. **C** The relative expression of CTF5 is detected using qRT-PCR following MCPIP1 overexpression and knockdown in MDA-MB-231 and MDA-MB-157 cells, respectively. **D** The relative expression of MCPIP1 and CTF5 is detected using qRT-PCR in xenografts. *GAPDH* is used as endogenous control. **E** Western blot analysis of MCPIP1 immunoprecipitates using anti-flag-tag antibody in MDA-MB-231 cells. Two replicates are performed. **F** MCPIP1-binding peak distribution across reference genomic regions. **G** Venn diagrams showing the overlap of MCPIP1-binding peak genes and RASGs. **H** IGV-sashimi plot of MCPIP1-binding across *NFIC*. **I** Quantification of NFIC expression by qRT-PCR using iRIP-seq data. **J** Motif analysis showed the top 5 preferred bound motifs of MCPIP1 in two replicate iRIP-seq samples using HOMER software. **K** Schematic diagram of the *NFIC* minigene constructs, showing both wild-type (WT) and the mutant (MT) in which CGGCCG was mutated to AGACCT. Boxes represent exons, lines between exons represent introns. **L** 293 T cells are co-transfected with vector or MCPIP1 overexpression plasmids and CTF5 wild-type minigene (WT) or CTF5 mutant minigene (MT) or control vector (Ctrl), semi-RT-PCR analysis is performed to detect the change in AS ratio (Ratio = AS splicing/model splicing). *GAPDH* is used as endogenous control. Error bars represent the mean ± SD from three independent experiments. *P < 0.05. ^ns^*P* ≥ 0.05.

Recently, emerging evidence has suggested that aberrant AS was involved in the tumorigenesis and development of breast cancer^38,39^. However, whether MCPIP1 exerts antitumor activity by modulating AS in TNBC is largely unknown. Our aforementioned RNA-seq data demonstrated that MCPIP1-RASGs are closely related to TNBC proliferation. Among these RASGs, *nuclear factor IC* (*NFIC*) has attracted attention, because its dysregulation was correlated with cell proliferation, migration, invasion, and even chemoresistance in a variety of solid tumors^40–42^. The splice variant formed by skipping exons 9 and 10 in the *NFIC* gene generates the 428-amino-acid-long CTF5 (ENST00000341919.7) (Fig. 4A), which displays the strongest transcriptional activation activity among the members of the NFIC family^43^. The ratio of the exons 9 and 10 skipping was increased after MCPIP1 overexpression based on our RNA-seq data, which was then confirmed by qRT-PCR (Fig. 4B), suggesting that MCPIP1 may promote the expression of CTF5. To further verify the role of MCPIP1 in regulating CTF5 expression, MCPIP1was overexpressed and silenced in MDA-MB-231 and MDA-MB-157 cells to detect CTF5 expression, respectively. qRT-PCR assay showed that MCPIP1 overexpression increased the expression of CTF5, whereas MCPIP1 knockdown resulted in decreased expression of CTF5 (Fig. 4C). Furthermore, we confirmed that enforced expression of MCPIP1 upregulated CTF5 expression in xenografts (Fig. 4D). Collectively, these results implied that CTF5 might participate in the MCPIP1-induced inhibition of TNBC cell proliferation.

As the initial study to explore MCPIP1-regulated AS in TNBC, we further investigated the association between MCPIP1 binding molecules and MCPIP1-regulated alternative splicing genes. To this end, we performed iRIP-seq and obtained a transcriptome-wide binding profile of MCPIP1 in MDA-MB-231 cells. We found that MCPIP1 selectively bound to gene regions including the CDS, 3′UTR, 5′UTR, and intron regions (Fig. 4E, F). Subsequently, we performed integrated analysis and found that 154 genes overlapped between MCPIP1-RASGs and MCPIP1 binding peaks in two replicates (Fig. 4G), suggesting MCPIP1 regulation of alternative splicing in these genes required MCPIP1-RNA binding. Exhilaratingly, the MCPIP1 binding peak was significantly enriched across *NFIC* (Fig. 4H). In addition, we confirmed MCPIP1 binding to *NFIC* using quantitative RIP-PCR in two replicate iRIP samples (Fig. 4I). Next, we ran HOMER algorithm to search for overrepresented motifs in MCPIP1-bounding peaks and found the over-presentation of CGGCCG motif in both replicated samples, suggesting CGGCCG motif might be the binding motif of MCPIP1 in *NFIC* pre-mRNA (Fig. 4J). To further verify that MCPIP1 regulates the skipping of exons 9 and 10 in the *NFIC*, we designed a minigene experiment. Two minigenes containing exons 8, 9, 10, 11 and flanking introns of *NFIC*, were constructed with normal CGGCCG (WT), and CGGCCG-mutated (MT) (Fig. 4K). Subsequently, we co-transfected MCPIP1 overexpression plasmid and *NFIC* minigenes into 293 T cells and examined the change in AS ratio. The results showed a significant increase in exon 9 and 10 skipping in constructed *NFIC* WT minigene in MCPIP1-overexpression cells compared with the vector group (Fig. 4l). Furthermore, when the MCPIP1 binding motif was mutated, no significant change in exon 9 and 10 skipping was observed between vector and MCPIP1-overexpression groups (Fig. 4L). Based on these results, we concluded that MCPIP1 directly bound the pre-mRNA of *NFIC* to regulate its exon skipping, promoting the production of CTF5 with higher transcriptional activity.

### CTF5 participates in the antiproliferative effect of MCPIP1 in TNBC

**Fig. 5:**
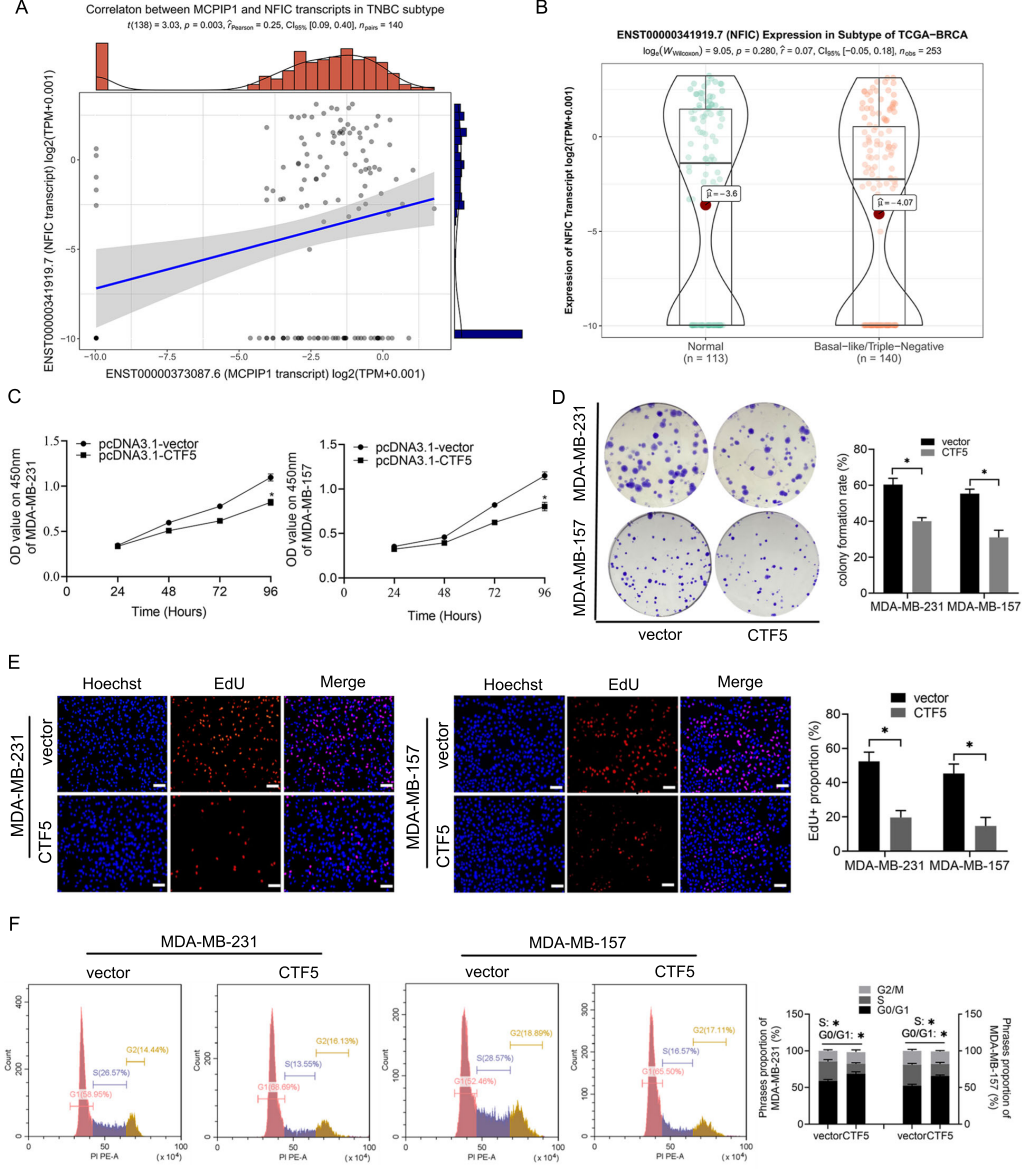
CTF5 induces cell cycle arrest and suppresses proliferation of TNBC cells. **A** The correlation analysis between MCPIP1 and CTF5 transcripts in TNBC samples from TCGA (*n* = 140). **B** Analysis of MCPIP1 mRNA levels in normal mammary tissues (*n* = 113) and TNBC tissues (*n* = 140) from TCGA database. **C** CCK8 assay is performed to examine the viability of MDA-MB-231 and MDA-MB-157 cells at 24, 48, 72 and 96 h following the indicated transfections. **D** Colony formation is performed following the indicated transfections. **E** EdU assay is performed following the indicated transfections to detect cell proliferation. Scale bars, 50 μm. **F** Cell cycle is detected using flow cytometry after the indicated transfections. Error bars represent the mean ± SD from three independent experiments. *P < 0.05.

Our experiments demonstrated that CTF5 was a direct target of MCPIP1, and there was a positive correlation between MCPIP1 and CTF5 expression in clinical TNBC samples from TCGA (Fig. 5A). We next examined CTF5 expression levels in TNBC by utilizing TCGA data. Although there was no significant difference in CTF5 expression between TNBC tissues and normal tissues, we observed a trend of reduction of CTF5 expression in TNBC tissues (Fig. 5B). Accordingly, these findings implied that CTF5 might be a negative regulator in TNBC. To investigate the role of CTF5 in TNBC cells, we transfected CTF5 overexpression plasmid into MDA-MB-231 and MDA-MB-157 cells. CCK-8, colony formation, and EdU assays showed that overexpression of CTF5 markedly hindered TNBC cell proliferation (Fig. 5C–E). Meanwhile, cell cycle analysis showed that elevated expression of CTF5 inhibited cell cycle transition from G0/G1 to S stage in TNBC cells (Fig. 5F).

Given that CTF5 functions as a tumor suppressor in TNBC, we next investigated whether CTF5 is responsible for MCPIP1-induced antiproliferative effect in TNBC. We silenced MCPIP1 and overexpressed CTF5 at the same time to examine whether re-expression of CTF5 could reverse the increased cell proliferation induced by MCPIP1 silencing. As expected, colony formation and EdU assays displayed that the acceleration in cell proliferation induced by MCPIP1 downregulation was inhibited by restoration of CTF5 expression (Supplementary Fig. 3A, B). Simultaneously, cell cycle analysis showed that re-expression of CTF5 abolished the promotion effect of MCPIP1-silencing on cell cycle progression (Supplementary Fig. 3C). Hence, these results revealed that CTF5 was a functional target of MCPIP1, and MCPIP1 suppressed proliferation of TNBC cells through upregulating CTF5.

### MCPIP1 downregulates cyclin D1-Rb-E2F1 axis via CTF5

**Fig. 6:**
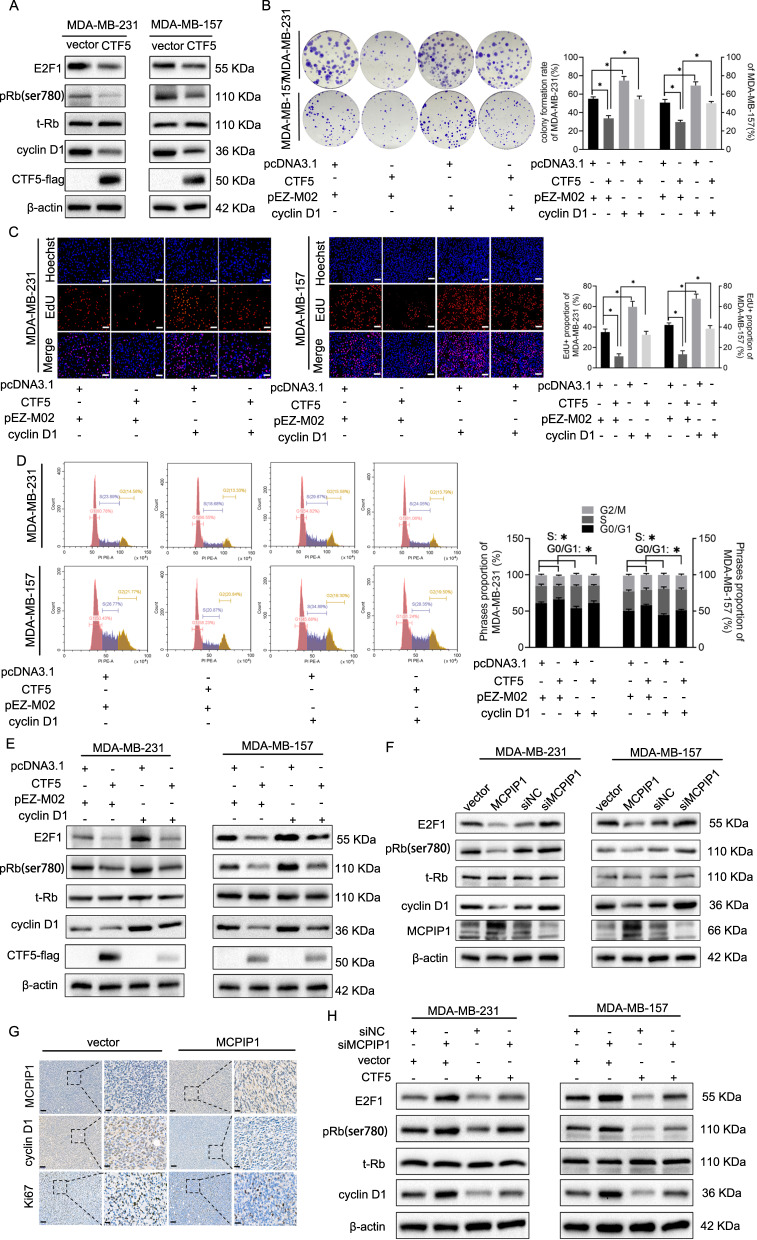
MCPIP1 inhibits cyclin D1-Rb-E2F1 axis through upregulating CTF5. **A** The relative expression of CTF5, cyclin D1, t-Rb, pRb and E2F1 is detected by western bot in MDA-MB-231 and MDA-MB-157 cells after transfected with control vector or CTF5 overexpression plasmid. β-actin is used as endogenous control. **B** Colony formation and EdU assays are performed to detect cell proliferation in MDA-MB-231 and MDA-MB-157 cells after indicated transfections. **C** EdU assay is performed following the indicated transfections. Scale bars, 50 μm. **D** Cell cycle is detected using flow cytometry after the indicated transfections. **E** The relative expression of CTF5, cyclin D1, t-Rb, pRb and E2F1 is detected using western blot following the indicated transfections. β-actin is used as internal control. **F** The expression of MCPIP1, cyclin D1, t-Rb, pRb and E2F1 is detected using western blot in MDA-MB-231 and MDA-MB-157 cells after indicated transfections. β-actin is used as internal control. **G** Immunohistochemical staining shows that overexpression of MCPIP1 inhibits the expression of cyclin D1 and Ki67 in xenografts. Scale bar, 20 and 100 μm. **H** The relative expression of cyclin D1, t-Rb, pRb and E2F1is detected using western blot in MDA-MB-231 and MDA-MB-157 cells after indicated transfections. β-actin is used as endogenous control. Error bars represent the mean ± SD from three independent experiments. *P < 0.05.

A previous study reported that NFIC could directly repress cyclin D1 transcription through binding to the promoter of cyclin D1 in breast cancer^42^. To explore whether CTF5 inhibits cyclin D1 expression, we quantified the expression level of cyclin D1 in CTF5-overexpressing TNBC cells. Cyclin D1 expression was notably decreased in CTF5-overexpressing cells, which was accompanied by the reduction in phosphorylated retinoblastoma (p-Rb) and E2F1 levels, the downstream signaling targets of cyclin D1. In contrast, the expression level of total Rb (t-Rb) remained unchanged (Fig. 6A). Furthermore, overexpression of cyclin D1 restored the impaired proliferation, stagnant cell cycle, and the decreased p-Rb and E2F1 levels (Fig. 6B–E). Interestingly, overexpression of MCPIP1 markedly downregulated cyclin D1 expression, while knockdown of MCPIP1 significantly upregulated cyclin D1 expression. Meanwhile, the expression of p-Rb and E2F1 also changed accordingly (Fig. 6F). Consistently, the result of IHC showed that enforced expression of MCPIP1 suppressed the expression of cyclin D1 and Ki67 in xenografts (Fig. 6G). Subsequently, we carried out a rescue experiment to confirm whether MCPIP1 regulates cyclin D1 expression in a CTF5-dependent manner. CTF5 overexpression plasmid and siRNA against MCPIP1 were co-transfected into MDA-MB-231 and MDA-MB-157 cells. Western blot assay showed that restoration of CTF5 resulted in reduced cyclin D1 accumulation induced by MCPIP1-knockdown. Meanwhile, the downstream signaling molecules p-Rb and E2F1 both showed a corresponding reduction (Fig. 6H). Collectively, these results suggested that MCPIP1 suppressed cyclin D1 expression by upregulating CTF5 levels, leading to cell cycle arrest at G0/G1 phase and ultimately inhibition of TNBC cell proliferation.

### CTF5 directly binds to cyclin D1 promoter to repress its transcription

**Fig. 7:**
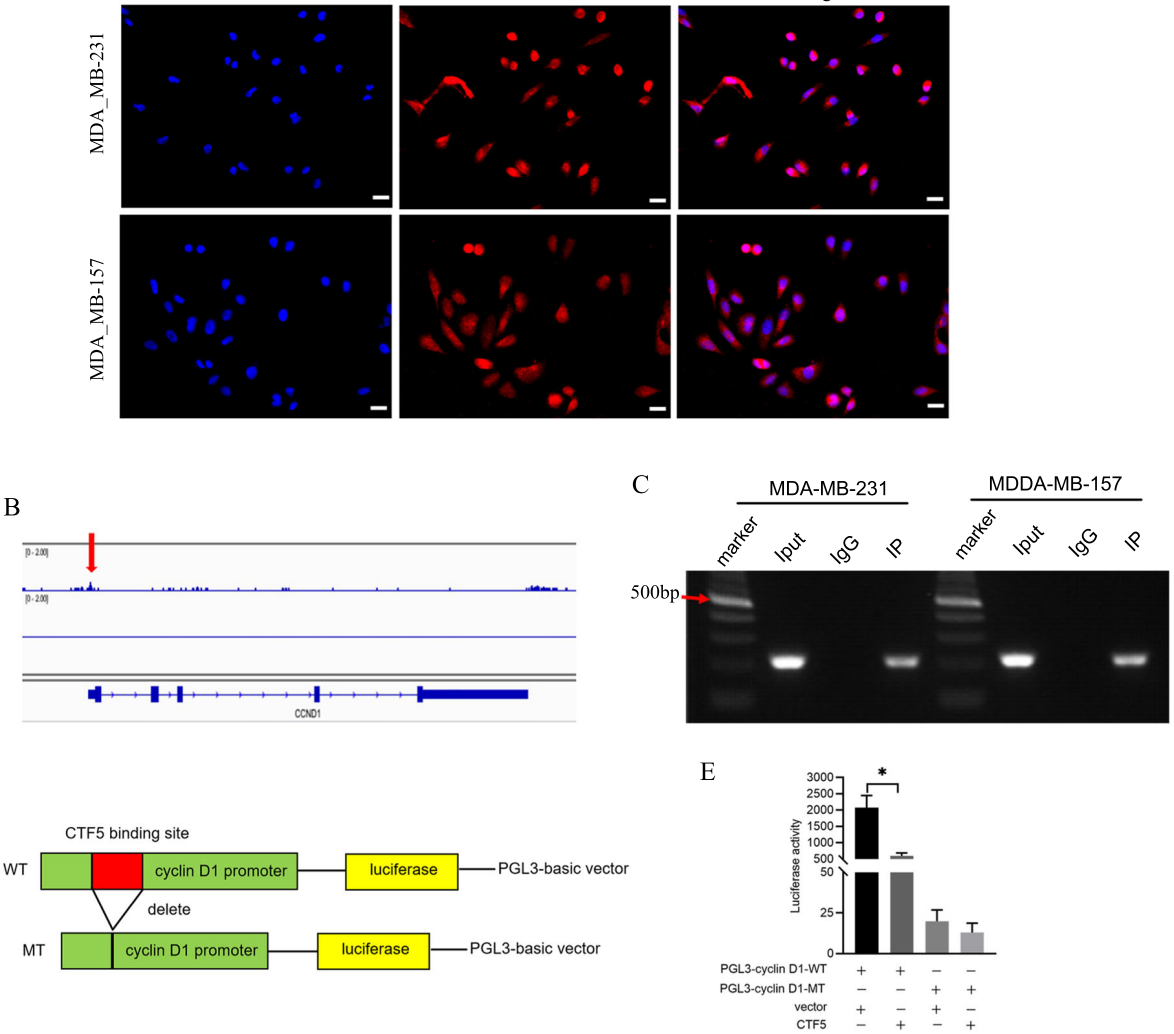
CTF5 directly binds to cyclin D1 promoter to repress its transcription. **A** Immunofluorescence assay shows that CTF5 is mainly localized in nucleus. scale bar, 20 μm. **B** One putative CTF5 binding site is predicted using IGV in cyclin D1 promoter, as pointed by red arrow. **C** ChIP assay shows the enrichment of CTF5 on the predicted promoter of cyclin D1 in MDA-MB-231 and MDA-MB-157 cells. Input DNA and IgG are used as positive and negative controls, respectively. **D** Schematic diagram of the luciferase reporter constructs incorporating wild-type cyclin D1 promoter and mutant cyclin D1 promoter in which the presumed CTF5 binding site is deleted. **E** Luciferase reporter assay is performed to detect the luciferase activity after co-transfected with PGL3-cyclin D1 (WT or MT) and CTF5 overexpression plasmid or control vector. Error bars represent the mean ± SD from three independent experiments. *P < 0.05.

Considering that CTF5 exhibited the strongest transcriptional activity of the NFIC family, we hypothesized that CTF5 downregulated cyclin D1 expression by directly repressing cyclin D1 transcription. To validate that CTF5 serves as a transcriptional repressor of cyclin D1, we first determined CTF5 subcellular localization. Immunofluorescence assay showed that CTF5 was mainly located in the nucleus of TNBC cells (Fig. 7A), suggesting the possibility of CTF5 was a transcription factor. In addition, we found that the cyclin D1 promoter harbored one potential CTF5 binding site (chr11:69455832–69456066) through the predictor tool Integrative Genomics Viewer (IGV)^44^ (Fig. 7B). The ChIP assay confirmed the significant enrichment of CTF5 in the predictive region of the cyclin D1 promoter (Fig. 7C). Finally, luciferase reporter assay showed that the luciferase activity of the cyclin D1 promoter was notably decreased in 293 T cells transfected with the CTF5 overexpression plasmid, whereas we did not detect significant luciferase activity of the cyclin D1 promoter when the CTF5 binding site was deleted (Fig. 7D, E). As the specific binding motif of CTF5 in the cyclin D1 promoter is undefined, we deleted the putative 235 nt CTF5-binding cluster, which might be the binding sites of most transcription factors. Consequently, we concluded that MCPIP1 inhibited cyclin D1 expression by upregulating CTF5 to directly repress cyclin D1 transcription.

## Discussion

TNBC is a highly aggressive malignancy that represents a refractory subtype of breast cancer^3^. The treatment of TNBC is always challenging due to the absence of effective therapeutic targets, leading to a finite response and resistance to existing treatment regimens^45,46^. Therefore, specific therapeutic targets need to be explored. Recently, the role of AS in human cancers has attracted attention. With the development of next-generation sequencing, we can comprehensively study AS on a genome-wide scale. The splicing patterns are frequently altered in cancer cells to generate cancer-specific isoforms, leading to a malignant transformation to acquire a more invasive phenotype, thereby driving cancer progression^47^. The process of AS is generally regulated by splicing factors, which are virtually RBPs and often dysregulated in cancers^21,22^. This means that a relatively small number of aberrant splicing factors can drive multiple oncogenic processes. Therefore, a comprehensive understanding of the mechanism of action of cancer-related splicing factors might facilitate development of a new class of anticancer therapeutics.

Recently, increasing evidence has suggested that the expression profile of splicing factors was aberrant in breast cancer^22^. These modifications resulted in many cancer-related genes undergoing aberrant ASEs to produce proteins with oncogenic properties^48,49^. In this study, we found that MCPIP1, a multifunctional RBP, was downregulated in TNBC tissues and cells in comparison with paired normal mammary tissues and cells. Importantly, high MCPIP1 expression was associated with prolonged OS. However, there were no significant correlations between MCPIP1 expression levels and the clinicopathological characteristics tested in our study. Collectively, these results suggested that MCPIP1 might be a reliable prognostic factor for TNBC patients.

Previous studies have demonstrated that MCPIP1 was a potent antioncogene in breast cancer, which could induce apoptosis by selectively degrading anti-apoptotic genes^28^, and inhibit migration and metastasis through suppressing TGF-β signaling^50^. However, the potential effect of MCPIP1 on the cell cycle in TNBC remains unclear. As cell cycle progression undertakes a crucial role in cell proliferation, deregulation of the cell cycle leads to uncontrolled proliferation that fuels tumorigenesis and cancer progression^51^. Here, we demonstrated that enforced expression of MCPIP1 induced cell cycle arrest at the G0/G1 stage, leading to decreased proliferation of TNBC cells both in vitro and in vivo. In summary, our study further confirmed the anti-oncogenic effect of MCPIP1 on TNBC, which might be a promising therapeutic target of TNBC.

Although MCPIP1 is an acknowleged RBP, the global effects of MCPIP1 on AS were uncharacterized prior to this study. Early studies of MCPIP1 antitumor roles in cancers have mainly focused on the RNase activity^28–30^. For example, in ccRCC, MCPIP1 inhibits tumor vascularization by suppressing the secretion of proangiogenic factors under hypoxic conditions, depending on the RNase activity^29^. Accordingly, to comprehensively elucidate the anti-oncogenic role of MCPIP1 in TNBC, we carried out RNA-seq and iRIP-seq and successfully demonstrated that MCPIP1 served as a splicing factor to broadly regulate ASEs in TNBC. Notably, MCPIP1-RASGs were highly correlated with cell cycle progression and proliferation of TNBC cells. Furthermore, we clarified that MCPIP1 promoted the production of CTF5 by regulating exons 9 and 10 skipping in *NFIC*. Several studies have demonstrated that NFIC acted as an antioncogene in multiple cancers, including esophageal squamous cell cancer^52^, bladder cancer^53^, breast cancer^54^. Nevertheless, the exact roles of CTF5 in TNBC still need to be elucidated. Our study indicated that enforced expression of CTF5 inhibited cell cycle progression and proliferation of TNBC cells, suggesting an antineoplastic role in regulating TNBC cell proliferation.

It is well documented that NFIC belongs the NFI family of site-specific transcription factors, which can modulate cancer-related genes transcription in various cancers^42,53–55^. Previously, NFIC was demonstrated to directly repress cyclin D1 transcription in breast cancer^42^. CTF5, a splicing variant of NFIC, has been revealed as the strongest transcriptional activator of the NFIC family^43^. Here, we demonstrated that CTF5 downregulated cyclin D1 expression by repressing its transcription. Cyclin D1, a well-known oncogene, is upregulated in up to 50% of breast cancers, which promotes G1–S cell cycle progression by forming a complex with cyclin dependent kinases 4/6 (CDK4/6), leading to the Rb phosphorylation and dissociation of transcription factor E2F from the pRb-E2F complex. Accordingly, the cyclin D1-Rb-E2F axis controls the transition through the G1/S cell cycle checkpoint^56^. Here, we observed that the downstream signaling molecules p-Rb and E2F1 were also suppressed by CTF5 overexpression.

Although our study confirmed that MCPIP1 exerted an antiproliferative role in suppressing cell cycle progression by regulating *NFIC* AS to promote the production of CTF5, thereby downregulating cyclin D1 expression, it remains unclear whether MCPIP1 suppresses cyclin D1 expression through other pathways such as RNase activity or deubiquitinase function. A recent study reported that overexpression of MCPIP1 caused G1/S checkpoint blockade, resulting in cell cycle arrest at G1 stage, which relies on the ribonuclease domain to reduce the key cyclins and CDKs involved in G1/S transformation in neuroblastoma cells^30^. Accordingly, further studies are required to explore these possibilities.

## Conclusions

**Fig. 8:**
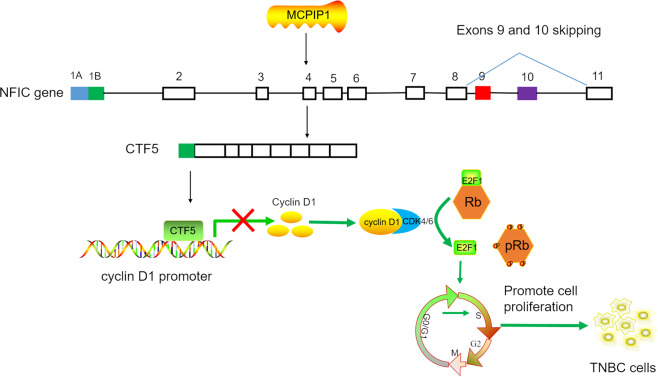
Schematic diagram of MCPIP1-regulated pathway in TNBC cells. MCPIP1 regulated *NFIC* exons 9 and 10 skipping to promote the production of CTF5 which downregulated cyclin D1-Rb-E2F1 axis to induce cell cycle arrest at G0/G1 stage, ultimately leading to inhibition of TNBC cell proliferation.

Our findings revealed that MCPIP1 was downregulated in TNBC tissues and cell lines, which exhibited an antiproliferative role through inducing cell cycle arrest at the G0/G1 stage. Furthermore, we found that high MCPIP1 expression levels were associated with better OS in TNBC patients. Notably, our study is the first to demonstrate that MCPIP1 extensively regulates AS in TNBC cells, which is highly associated with cell cycle progression and proliferation of TNBC cells. Finally, we found that MCPIP1 suppressed cell cycle progression by regulating *NFIC* exons 9 and 10 skipping to promote the production of CTF5, which is a transcriptional repressor of cyclin D1 to directly downregulate cyclin D1 expression accompanied by the reduction of downstream signaling targets p-Rb and E2F1, ultimately leading to inhibition of TNBC cell proliferation (Fig. 8). Therefore, our findings provide novel insights into the anti-oncogenic role of MCPIP1 in TNBC suggesting that MCPIP1 might be a reliable prognostic factor as well as a potential therapeutic target of TNBC.

## Supplementary information

supplementary figure 1

supplementary figure 2

supplementary figure 3

supplementary table 1

supplementary table 2

supplementary table 3

## Data Availability

The datasets and R-code used and/or analyzed during the current study are available from the corresponding author on reasonable request.
