# Supplementary material for: MCPIP1-mediated NFIC alternative splicing inhibits proliferation of triple-negative breast cancer via cyclin D1-Rb-E2F1 axis
Source: Cell Death Dis. 2021 Apr 6;12(4):370. doi: 10.1038/s41419-021-03661-4 (PMC8024338; doi:10.1038/s41419-021-03661-4)
Supplement: Supplementary file 1 — supplementary figure 1 [file 41419_2021_3661_MOESM1_ESM.docx]

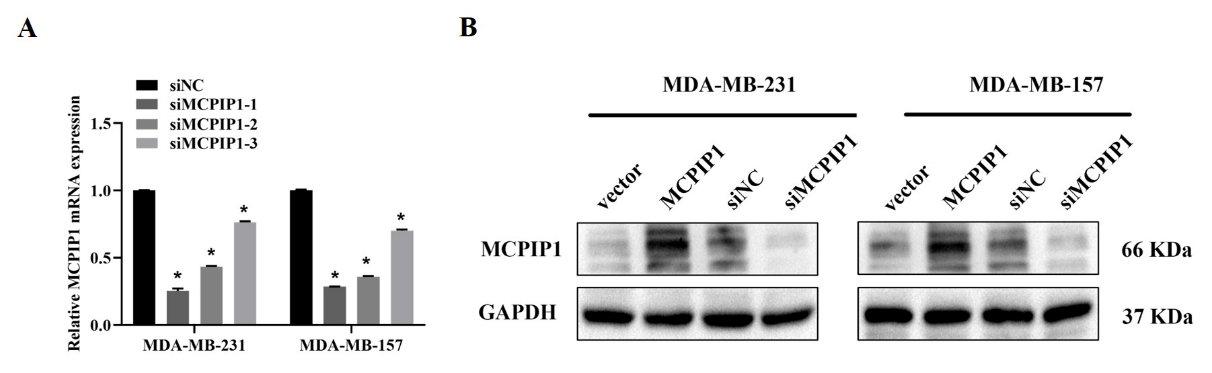


**Supplementary figure 1 Detection the efficiency of MCPIP1 silencing and overexpression in MDA-MB-231 and MDA-MB-157 cells. A** The relative expression of MCPIP1 is detected using qRT-PCR after transfected with siRNA scramble and three different MCPIP1 siRNA duplexes. *GAPDH* is used as endogenous control. **B** The relative expression of MCPIP1 is detected using western blot after indicated transfections. *GAPDH* is used as endogenous control. Error bars represent the mean ± SD from three independent experiments. **P* < 0.05.
