# Supplementary material for: MCPIP1-mediated NFIC alternative splicing inhibits proliferation of triple-negative breast cancer via cyclin D1-Rb-E2F1 axis
Source: Cell Death Dis. 2021 Apr 6;12(4):370. doi: 10.1038/s41419-021-03661-4 (PMC8024338; doi:10.1038/s41419-021-03661-4)
Supplement: Supplementary file 2 — supplementary figure 2 [file 41419_2021_3661_MOESM2_ESM.docx]

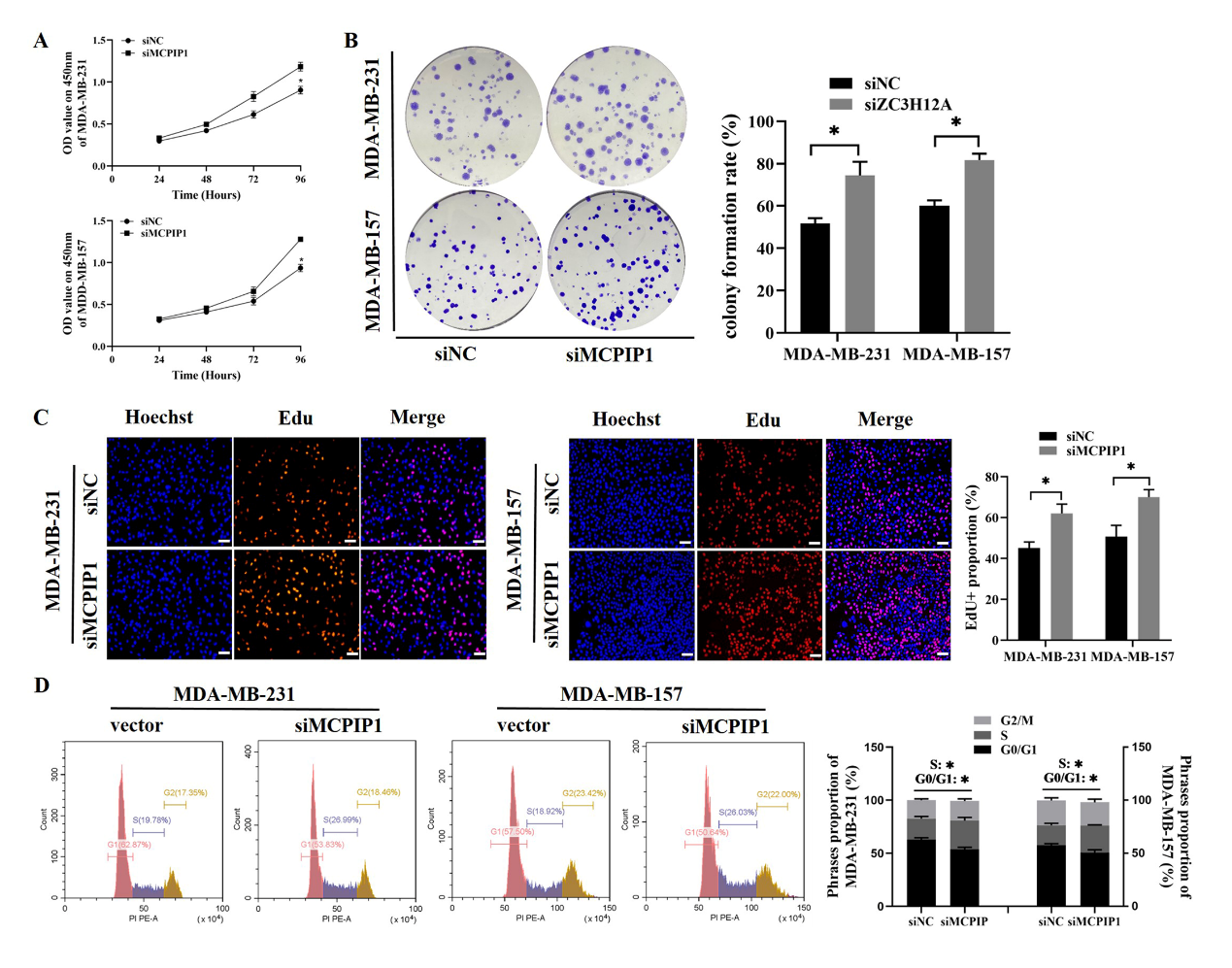


**Supplementary figure 2 MCPIP1 inhibits cell cycle progression and proliferation of TNBC cells.** **A** MDA-MB-231 and MDA-MB-157 cells are transfected with the siRNA scramble or siRNA against MCPIP1. CCK8 assay is performed to examine cell viability at 24, 48, 72, and 96 h. **B** Colony formation is performed after the indicated transfections. **C** EdU assay is performed following the indicated transfections to detect cell proliferation. Scale bars, 50 μm. **D** Cell cycle is detected using ﬂow cytometry after the indicated transfections. Error bars represent the mean ± SD from three independent experiments. **P* < 0.05.
