# Supplementary material for: MCPIP1-mediated NFIC alternative splicing inhibits proliferation of triple-negative breast cancer via cyclin D1-Rb-E2F1 axis
Source: Cell Death Dis. 2021 Apr 6;12(4):370. doi: 10.1038/s41419-021-03661-4 (PMC8024338; doi:10.1038/s41419-021-03661-4)
Supplement: Supplementary file 3 — supplementary figure 3 [file 41419_2021_3661_MOESM3_ESM.docx]

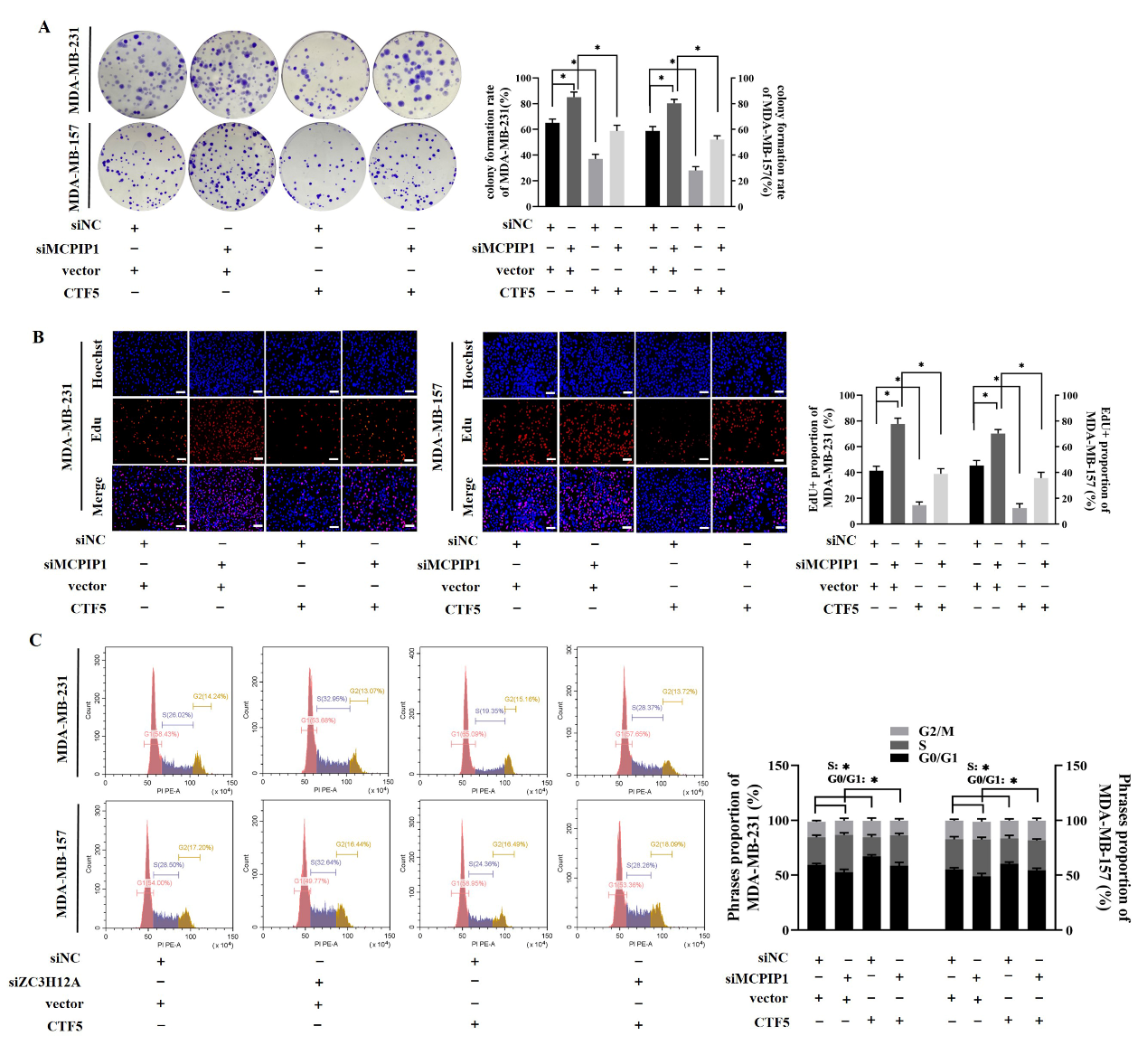


**Supplementary figure 3 CTF5** **is responsible for MCPIP1-induced antiproliferative effect in TNBC.** **A** MDA-MB-231 and MDA-MB-157 cells are co-transfected with control vector or CTF5 overexpression plasmid, and siRNA scramble (control) or siRNA against MCPIP1, colony formation assay is performed to test cell viability. **B** EdU assay is performed following the indicated transfections to detect cell proliferation. Scale bars, 50 μm. **C** Cell cycle is detected using ﬂow cytometry following the indicated transfections. Error bars represent the mean ± SD from three independent experiments. **P* < 0.05.
