# Supplementary material for: MCPIP1-mediated NFIC alternative splicing inhibits proliferation of triple-negative breast cancer via cyclin D1-Rb-E2F1 axis
Source: Cell Death Dis. 2021 Apr 6;12(4):370. doi: 10.1038/s41419-021-03661-4 (PMC8024338; doi:10.1038/s41419-021-03661-4)
Supplement: Supplementary file 4 — supplementary table 1 [file 41419_2021_3661_MOESM4_ESM.docx]

Supplementary Table 1: Association between MCPIP1 expression and clinicopathological characteristics of 80 TNBC patients

|  | MCPIP1 expression | | | | P value |
| --- | --- | --- | --- | --- | --- |
| parameters | Low (n=51) | % | High (n=29) | % |  |
| Age | | | | | 0.227 |
| ≤50 years | 30 | 69.8 | 13 | 30.2 |  |
| >50 years | 21 | 56.8 | 16 | 43.2 |  |
| Grade | | | | | 0.981 |
| Low | 28 | 63.6 | 16 | 36.4 |  |
| High | 23 | 63.9 | 13 | 36.1 |  |
| Tumor size | | | | | 0.291 |
| ≤5 cm | 47 | 61.8 | 29 | 38.2 |  |
| >5cm | 4 | 100 | 0 | 0 |  |
| Lymph node metastasis |  | | | | 0.408 |
| Negative | 25 | 59.5 | 17 | 40.5 |  |
| Positive | 26 | 68.4 | 12 | 31.6 |  |
| TNM staging |  |  |  |  | 0.593 |
| Ⅰ-Ⅱ | 38 | 65.5 | 20 | 34.5 |  |
| Ⅲ-Ⅳ | 13 | 59.1 | 9 | 40.9 |  |

*p* < 0.05 *represents statistical significance (Chi-square test).*
