# Supplementary material for: MCPIP1-mediated NFIC alternative splicing inhibits proliferation of triple-negative breast cancer via cyclin D1-Rb-E2F1 axis
Source: Cell Death Dis. 2021 Apr 6;12(4):370. doi: 10.1038/s41419-021-03661-4 (PMC8024338; doi:10.1038/s41419-021-03661-4)
Supplement: Supplementary file 5 — supplementary table 2 [file 41419_2021_3661_MOESM5_ESM.docx]

Supplementary Table 2: primer sequences

| **Primers for PCR** |  |
| --- | --- |
| MCPIP1-Forward | ACGGGATCGTGGTTTCCAAC |
| MCPIP1-Reverse | TGGCTTCTTACGCAGGAAGTT |
| Cyclin D1-Forwrd | GCTGCGAAGTGGAAACCATC |
| CyclinD1-Reverse | CCTCCTTCTGCACACATTTGAA |
| CTF5-Forward | ACCTGGACCGTCCTGGTATC |
| CTF5-Reverse | GGTGTTCTTGTTCACTCTAA |
| GAPDH-Forward | GGAGCGAGATCCCTCCAAAAT |
| GAPDH-Reverse | GGCTGTTGTCATACTTCTCATGG |
| CHIP-PCR  cyclin D1-Forward | GGCTTTGATCTTTGCTTAACA |
| CHIP-PCR  cyclin D1-Reverse | TCCGCGCTCGGCTCTCGCTT |
| NFIC-ES Model-Forward | TCAGGCACAGTCCTGGTATC |
| NFIC-ES Model-Reverse | GGTGTTCTTGTTCACTCTAA |
| NFIC-ES AS-Forward | ACCTGGACCGTCCTGGTATC |
| NFIC-ES AS-Reverse | GGTGTTCTTGTTCACTCTAA |
| Minigene-semi-RT-PCR  NFIC-Forward | CGTCCTCCGCTCTGCATTT |
| Minigene-semi-RT-PCR  NFIC-Reverse | CAGGCGGAGAGGAGATGAATA |
| RIP-PCR  NFIC-Forward | CCATCCGCTACCCACCTCAT |
| RIP-PCR  NFIC-Reverse | GCAGGCCAGCGAGACAAGAT |
|  |  |
| **The sequences of siRNA targeting MCPIP1** |  |
| siNC sense | 5’-UUCUCCGAACGUGUCACGUTT-3’ |
| siNC antisense | 5’-ACGUGACACGUUCGGAGAATT-3’ |
| siMCPIP1#1 sense | 5’-CCGAGAUCCUCUCCUACAATT-3’ |
| siMCPIP1#1 antisense | 5’-UUGUAGGAGAGGAUCUCGGTT-3’ |
| siMCPIP1#2 sense | 5’-GGGUCUAACACAGACCUAUTT-3’ |
| siMCPIP1#2 antisense | 5’-AUAGGUCUGUGUUAGACCCTT-3’ |
| siMCPIP1#3 sense | 5’-GGUCUGAACCAUACCCACUTT-3’ |
| siMCPIP1#3 antisense | 5’-AGUGGGUAUGGUUCAGACCTT-3’ |
|  |  |
| **Primers for plasmid construction** |  |
| Forward primers for cyclin D1 promoter (mutant) | TATGTGACTGTCTTGCGCCCGGGATGG |
| Reverse primers for cyclin D1 promoter (mutant) | TACAAGCTTCAGAGAATGGGAGCGGGAG |
