# Supplementary material for: MCPIP1-mediated NFIC alternative splicing inhibits proliferation of triple-negative breast cancer via cyclin D1-Rb-E2F1 axis
Source: Cell Death Dis. 2021 Apr 6;12(4):370. doi: 10.1038/s41419-021-03661-4 (PMC8024338; doi:10.1038/s41419-021-03661-4)
Supplement: Supplementary file 6 — supplementary table 3 [file 41419_2021_3661_MOESM6_ESM.docx]

Supplementary Table 3: Classification of all MCPIP1 RASEs between MCPIP1

overexpression and control vector groups

| Event Type | schematic diagram  of ASE | | ZC3H12A_vs_Ctrl  Up | | | ZC3H12A_vs_Ctrl  Down |
| --- | --- | --- | --- | --- | --- | --- |
| 3pMXE | 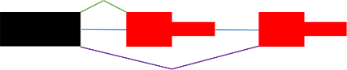 | | 5 | | | 10 |
| 5pMXE | | 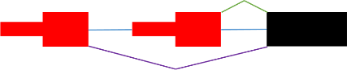 | 13 | | | 9 |
| A3SS | | 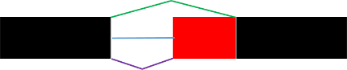 | 61 | | | 66 |
| A3SS  &ES | | 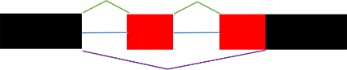 | 8 | | | 8 |
| A5SS | | 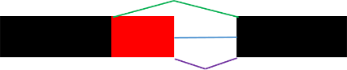 | 67 | | | 87 |
| A5SS  &ES | | 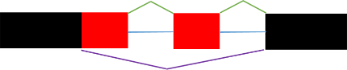 | 7 | | | 10 |
| ES | | 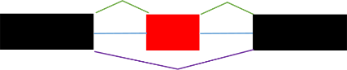 | 58 | | | 71 |
| IR | | 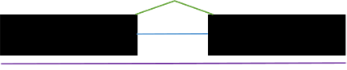 | 87 | | | 65 |
| MXE | | 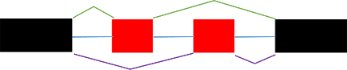 | 12 | | | 14 |
| Cassette Exon | | 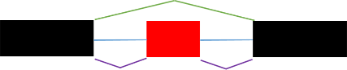 | | 45 | | 59 |
| Total |  | | | | 363 | 399 |

Notes: boxes represent exons, blue lines represent introns, green line represents model splicing, purple line represent alternative splicing
